# Planted Citrus Regulates the Community and Networks of *phoD*-Harboring Bacteria to Drive Phosphorus Availability Between Karst and Non-Karst Soils

**DOI:** 10.3390/microorganisms12122582

**Published:** 2024-12-13

**Authors:** Xuan Yu, Lulu Feng, Yuan Huang, Yueming Liang, Fujing Pan, Wei Zhang, Yuan Zhao, Yuexin Xiao

**Affiliations:** 1 Guangxi Key Laboratory of Theory and Technology for Environmental Pollution Control, College of Environmental and Engineering, Guilin University of Technology, Guilin 541004, China; 2 Guangxi Botanical Garden of Medicinal Plants, Nanning 530023, China; 3 Karst Dynamics Laboratory, Ministry of Natural Resources & Guangxi, Institute of Karst Geology, Chinese Academy of Geological Sciences, Guilin 541004, China; 4 Key Laboratory of Agro-Ecological Processes in Subtropical Region, Institute of Subtropical Agriculture, Chinese Academy of Sciences, Changsha 410125, China; 5 Huanjiang Agriculture Ecosystem Observation and Research Station of Guangxi, Guangxi Key Laboratory of Karst Ecological Processes and Services, Huanjiang Observation and Research Station for Karst Ecosystems, Chinese Academy of Sciences, Huanjiang 547100, China; 6 Changsha Comprehensive Survey Center of Natural Resources, China Geological Survey, Changsha 410600, China

**Keywords:** cultivation, lithology, P availability, *phoD*-harboring bacteria, network connectivity

## Abstract

The phosphorus (P) availability in soils is influenced by microbes, particularly those containing the gene responsible for phosphate solubilization. The present study investigated the community structure, diversity, and co-occurrence networks of *phoD*-harboring bacteria in karst and non-karst citrus orchard soils across a planting duration gradient, natural forests, and abandoned land, as well as the soil total P (TP) and available P (AP) contents and enzyme activities. The soil AP contents were lower in the karst regions than in the non-karst regions, while the soil organic carbon (C; SOC), exchangeable calcium, and microbial biomass nitrogen (N) contents; alkaline phosphatase (ALP) and β-Glucuronidase activities; and pH had the opposite trends. In addition, the soil AP and SOC contents and the ALP and acid phosphatase (ACP) activities in the karst regions decreased with an increase in the planting years, whereas the AP, TP, and microbial biomass P contents and ACP activities in the non-karst regions increased. The diversity indices and network complexity of *phoD*-harboring bacteria were higher in the karst regions than in the non-karst regions, with marked community differences between different planting years in the non-karst regions. The soil AP was significantly and positively correlated with the rare genera *Pelagicola*, *Methylobacter*, *Streptomyces*, and *Micromonospora* in the karst regions and *Roseivivax*, *Collimonas*, *Methylobacterium*, *Ralstonia*, and *Phyllobacterium* in the non-karst regions. Structural Equation Modeling showed that citrus cultivation altered the soil pH, SOC, and total N, and, in turn, the *phoD*-harboring bacterial community structure and diversity, which led to changes in the ALP activity and P availability. Thus, the rare genera of the *phoD*-harboring bacteria, influenced by the pH and SOC, highly regulated the availability of P in the karst and non-karst citrus orchard soils.

## 1. Introduction

Phosphorus (P) is a vital nutrient for plant growth in terrestrial ecosystems, particularly in artificial ecosystems (e.g., farmland and orchards) [1]. Generally, farmers apply high levels of phosphate fertilizer into orchards to increase the crop yield. For example, a high P gradient ranging from 100 to 200 kg ha^−1^ is suggested for the fertilization of citrus orchards globally [2], with an average of 198 kg ha^−1^ applied in China [3]. However, approximately 42–54% of the P fertilizer applied in farmland soils is not utilized [4,5], resulting in leaching into surface water and groundwater systems, in addition to the eutrophication of water bodies [6]. The soil type, crop planting year, and microbial communities are the key drivers of soil P availability [7,8,9]. However, their potential interactions with soil P availability in citrus orchards in karst and non-karst regions remain unclear. Thus, examining the interactions between soil type, planting year, and microorganisms that drive soil P availability is necessary, which could facilitate the appropriate regulation of P in orchards.

The availability of P in soil is dynamic and involves the transformation of both inorganic and organic P fractions. Such a transformation is influenced by the soil type and soil microbial activity [10]. In general, P is incorporated readily into inorganic fractions, such as iron phosphorus (Fe-P), aluminum phosphorus (Al-P), and calcium-phosphorus (Ca-P), which are associated with the presence of iron, aluminum, and calcium [11]. Some soil microbes can produce alkaline and acid phosphatases [12], which are encoded by *phoA*, *phoD*, and *phoX* genes. For instance, *phoD*-harboring bacteria can exude alkaline phosphatase (ALP) by encoding the *phoD* gene, thereby facilitating the conversion of organic P into forms that are more accessible to plants and crops [13]. Long-term fertilization has been reported to suppress the expression of the *phoD* gene and reduce both the abundance and diversity of *phoD*-harboring bacteria, as well as their topological coefficients in co-occurrence networks [8]. However, declines in the accumulation, dissolution, and effective utilization of soil P will stimulate the *phoD* gene expression by these microbes [14]. A few studies showed that the rare taxa of *phoD*-harboring bacteria have a more significant role in soil available P (AP) increases than the dominant species [15,16]. However, limited information is available regarding the relationships between the *phoD*-harboring bacterial taxa and the soil AP in both karst and non-karst citrus orchards.

The karst landform in southwest China is one of the largest karst regions globally, covering an area of approximately 5.4 × 10^5^ km^2^ [17]. The bedrock of karst farmlands is highly exposed, resulting in poor water and poor soil conservation capacity. In addition, the soils exhibit high levels of Ca and magnesium (Mg) and possess a relatively high buffering capacity against acidification [18]. Compared with the calcareous soil in karst regions, non-karst regions typically harbor acidic soils containing Fe-P and Al-P at the same latitude [19]. Citrus production in China was projected to reach 6.0 × 10^7^ t by 2022. Out of the total output, Guilin alone would contribute approximately 6.9 × 10^6^ t. Extensive citrus cultivation regions are distributed across both karst and non-karst regions within the Li River basin in Guilin. The associated widespread fertilizer application is expected to influence soil P speciation and transformation. Although some studies demonstrated that soil P availability is influenced by the soil type, planting years, and microbial communities [8,10,16,18], the influence of *phoD*-harboring bacteria on P availability and transformation in citrus orchards across different planting years in karst and non-karst regions remain unclear.

The present study assessed the community structure, diversity, and co-occurrence network of soil *phoD*-harboring bacteria in karst and non-karst citrus soils across three planting durations (5, 10, and 15 years) and in control sites (natural forest land and abandoned land). Additionally, the soil total P (TP) and AP; acid phosphatase (ACP) and ALP activities; and microbial biomass carbon (C; MBC), microbial biomass nitrogen (N; MBN), and microbial biomass P (MBP), and soil organic C (SOC) contents were analyzed. The authors hypothesized that (1) the soil AP is lower in karst citrus orchards than in non-karst citrus orchards; (2) the diversity, richness, and network complexity of *phoD*-harboring bacteria are higher in karst citrus orchard soils across different planting durations than in non-karst regions; and (3) rare *phoD*-harboring bacterial taxa exert a greater influence on P availability than dominant species. The findings of the present study could enhance our understanding of soil P dynamics, *phoD*-harboring bacterial community structure, and their interactions in both karst and non-karst regions, which could facilitate the exploitation of *phoD*-harboring bacteria to enhance the P availability in orchards cultivated in such regions.

## 2. Materials and Methods

### 2.1. Study Site

**Figure 1:**
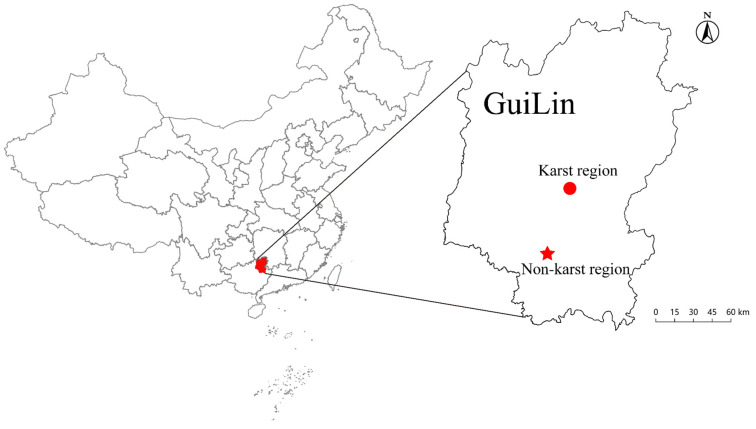
Citrus sampling sites in karst and non-karst regions of Guilin, southwestern China.

The present study was carried out in the karst and non-karst regions of the Lijiang River Basin within Guilin (24°48′~25°24′ N, 110°22′~110°19′ E), Guangxi Zhuang Autonomous Region, southwestern China (Figure 1). Both regions are located in the subtropical monsoon zone, featuring an average annual temperature of around 18 °C and an annual rainfall of 1915.2 mm. The period from April to July is the most rainfall-abundant season, accounting for over 65% of the total annual precipitation [20]. According to the FAO/UNESCO classification system, soils in the karst and non-karst regions are classified as lithosols and ferralsols, respectively [21].

### 2.2. Sampling

The three ages of citrus orchards were 5 years (Y5), 10 years (Y10), and 15 years (Y15). These sites had consistent fertilization levels, receiving an annual application of 200 kg ha^−1^ y^−1^ NPK compound fertilizer (N–P_2_O_5_–K_2_O, 15:15:15) and 3000 kg ha^−1^ y^−1^ of organic fertilizer (pigeon droppings, 4 kg N ha^−1^ y^−1^, 3 kg P ha^−1^ y^−1^, 3 kg K ha^−1^ y^−1^). The NPK compound fertilizer was applied from March to September, while the organic fertilizer was mainly used in January to promote fruit swelling. Pesticides were applied based on the growth of the fruit tree and the pest situations. The number of applications per year varied from 14 to 18, with a dosage of 6 L ha^−1^ for each application. Simultaneously, natural forest (NF) and abandoned land (AL) were selected as control groups to assess the alterations in the soil P cycling microbial community diversity and the microbial community structure induced by the citrus cultivation.

Soil sampling was conducted from July to August 2022. There were five replicate plots (20 m × 20 m, with similar slope directions and gradients) in each planting age stage, and the distance between the plots was not less than 30 m. In total, 50 plots (2 lithological types × 5 stages × 5 replicates) were established, and 50 soil samples were collected by combining five replicate soil sub-samples (20 cm depth) at each plot. Each sieved soil sample was divided into three parts: two sub-samples were stored at 4 °C and −80 °C for use in the determination of the soil enzyme activity and the microbial biomass and *phoD* gene sequencing, respectively; the third subsample was air-dried and used for the soil physical and chemical property analyses.

### 2.3. General Soil Parameters

The methods for the determination of pH; SOC; TN; TP; AP; exchangeable Ca (ExCa^2+^); exchangeable magnesium (ExMg^2+^); and microbial biomass C (MBC), N (MBN), and P (MBP) are described in our previous study [22]. The activities of β-Glucosidase (βG), ACP, and ALP were measured using a fluorescence spectrophotometer (Infinite M200 PRO, Tecan, Switzerland) [23].

### 2.4. DNA Extraction and Illumina Sequencing

DNA was isolated from 0.5 g frozen soil using a FastDNA SPIN kit (MP Biomedicals, Cleveland, OH, USA) according to the extraction protocol provided by the manufacturer. Qualitative and quantitative analyses of the total DNA in the soil microorganisms were performed using 1% (*w*/*v*) agarose gel electrophoresis and UV spectrophotometry (NanoDrop Technologies, Wilmington, NC, USA), respectively.

*phoD* was amplified using the primers ALPS-F730 (5′-CAGTGGGACGACCACGAGGT-3′) and ALPS-1101 (5′-GAGGCCGATCGGCATGTCG-3′) [24]. Each sample was amplified in triplicate within a 25 μL reaction: 2.5 μL of 10× Ex Taq Buffer (Mg^2+^ plus), 0.3 μL of Ex Taq (Takara Biotechnology, Dalian Co., Ltd., Dalian, China), 1 μL (10 pM) of each forward and reverse primer, 1 μL (~30 ng) of DNA template, and the rest of the volume was filled with ddH_2_O. The protocol for amplification involved the following steps: initial denaturation at 94 °C for 5 min, followed by 30 cycles of 94 °C for 30 s, annealing at 52 °C for 30 s, and extension at 72 °C for 30 s. The final extension step was performed at 72 °C for an additional 10 min.

After the PCR amplification, the product DNA was purified using an E.Z.N.A.^®^ Gel Extraction Kit (Omega, Mountain Lakes, NJ, USA) to enhance the purity and yield of the target sequence. Purified DNA was sequenced on the Illumina Nova 6000 platform (Guangdong Magigene Biotechnology Co., Ltd., Guangzhou, China). The raw sequences were processed using the QIIME 2 platform [25]. Sequences that contained ambiguous bases shorter than 200 bp and those with average quality scores < 20 were excluded. Sequences with chimeras were removed using USEARCH on the QIIME 2 platform. The remaining nucleotide sequences that did not correspond to amino acid sequences for *phoD* or had a termination codon were removed using the FrameBot tool in the RDP function gene pipeline. The obtained high-quality sequences were clustered into OTUs using UCLUST at 97% similarity. Subsequently, the taxonomic assignment of each OTU was performed using BLAST from the Greengenes database [26]. The BioProject accession number of the sequence data is PRJNA1105464 in the NCBI database.

### 2.5. Statistical Analyses

To construct the microorganic co-occurrence networks in the citrus orchards in the karst and non-karst regions, the OTUs of *phoD*-harboring bacteria present in <0.01% of the samples were first excluded to reduce bias [27]. Co-occurrence networks were constructed using “Gephi 0.9.2” based on Spearman’s correlations with an r > 0.6 and *p* < 0.05, following a Benjamini–Hochberg correction. The topological characteristics of the network model included the total numbers of nodes and links, average path lengths, and clustering coefficients [28]. The nodes were categorized according to their within-module connectivity (Zi) and between-module connectivity (Pi), which facilitated the differentiation of nodes based on their topological roles within the network. The node topologies were categorized into four categories: module hubs (nodes with high connectivity within modules, Zi > 2.5), network hubs (nodes with high connectivity across the entire network, Zi > 2.5 and Pi > 0.62), connectors (nodes that bridged different modules, Pi > 0.62), and peripherals (nodes with limited external connections within modules, Zi < 2.5 and Pi < 0) [29]. Network hubs, module hubs, and connectors are considered keystone taxa because of their pivotal influence on the structure and the potential functional significance within microbial communities [30]. Subsequently, using the “Hmisc” package in R software (R v4.3.1), a correlation network diagram that integrated the soil P, soil physicochemical properties, and *phoD*-harboring bacteria at the genus level was drawn [31].

The normal distribution of each index was verified. The Least Significant Difference test was used to investigate the differences in the soil physicochemical attributes, phosphatase activity, and *phoD*-harboring bacteria diversity across different planting years in the karst and non-karst regions. Principal Coordinate Analysis (PCoA) was used to visualize the differences in the community structures of the *phoD*-harboring bacteria between planting years in the two regions. The Mantel test was performed to examine the relationships between the Richness, Chao 1, Shannon–Wiener, Simpson, and Evenness diversity indices, as well as the *phoD*-harboring bacterial community structure, soil nutrients, and ALP and ACP activities. The Random Forest model (“randomForest” package in R software (R v4.3.1)) was used to assess the relative importances of various parameters on the soil AP and *phoD*-harboring bacterial community structure. The relative importances of the measured variables were evaluated based on the Mean Squared Error (MSE%). Finally, Structural Equation Modeling (SEM) was used to test the causal relationships between the ALP, pH, AP, SOC, and TN and the *phoD*-harboring bacteria relative abundance. The best-fitting model was derived using the maximum likelihood method based on model fit, which included the chi-square test (χ2), Goodness-of-Fit Index, and Root Mean Square Error of Approximation.

## 3. Results

### 3.1. Soil P Contents and Enzyme Activities in Karst and Non-Karst Citrus Cultivations

**Figure 2:**
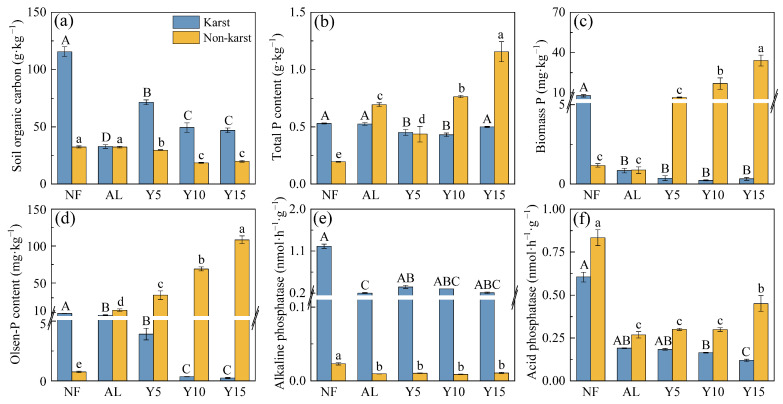
The contents of soil organic carbon (SOC) (**a**), total P (TP) (**b**), microbial biomass P (MBP) (**c**), available P (AP) (**d**), as well as the activities of alkaline phosphatase (ALP) (**e**) and acid phosphatase (ACP) (**f**), in citrus orchards of different cultivation ages in the karst and non-karst regions. Different capital and lowercase letters indicate significant differences between different citrus plantation ages in karst and non-karst regions (*p* < 0.05), respectively. NF, natural forest; AL, abandoned land; Y5, 5-years planting; Y10, 10-years planting; Y15, 15-years planting.

The SOC content and ALP activity were higher in the karst regions than in the non-karst regions (Figure 2a,e); however, the ACP activity exhibited the opposite trends (Figure 2f). The TP, MBP, and AP contents in the natural forest land were higher in the karst regions than in the non-karst regions; however, the values for the three planting ages and abandoned lands were lower in the karst regions than in the non-karst regions (Figure 2b–d). In the karst regions, the SOC and AP contents and the ALP activity were much higher in the natural forests than in the cultivated and abandoned lands, where the SOC, MBP, and AP contents decreased with an increase in the planting years. In the non-karst regions, the ALP and ACP activities were much higher in the natural forests than in the cultivated and abandoned lands, where the TP, MBP, and AP contents and the ACP activity increased, whereas the SOC decreased, with an increase in the planting years.

### 3.2. Community Structure, Diversity, and Co-Occurrence Networks of phoD-Harboring Bacteria

**Figure 3:**
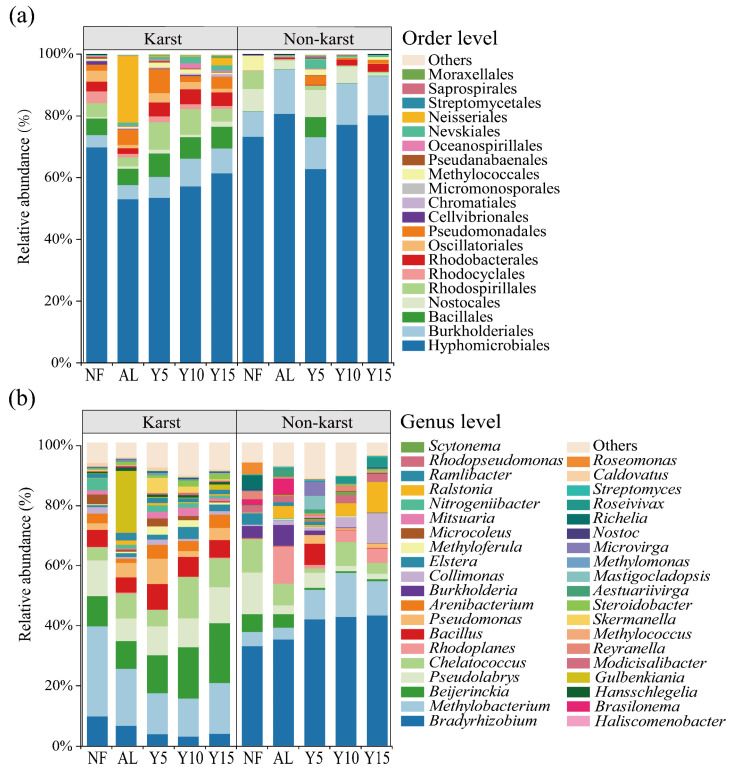
The relative abundance of *phoD*-harboring bacteria at order (**a**) and genus (**b**) levels in citrus orchards of different ages within karst and non-karst regions. NF, natural forest; AL, abandoned land; Y5, 5-years planting; Y10, 10-years planting; Y15, 15-years planting.

After the quality filtering of the Illumina data, the sequencing for the karst and non-karst regions yielded altogether 238,138 and 327,415 high-quality sequences, respectively. The average numbers of OTUs were 1700 and 843 in the citrus soils from the karst and non-karst regions, respectively. At the order level (>4%), Hyphomicrobiales was the dominant taxon (53.12~69.93% in all five stages) of the *phoD*-harboring microbes in citrus soils from the karst region (Figure 3a), followed by Burkholderiales (4.00~8.98% in all five stages) and Bacillales (5.29~7.01% in all five stages). In the non-karst citrus soils, Hyphomicrobiales was also the dominant taxon (62.82~80.75% in all five stages), followed by Burkholderiales (8.25~14.41% in five stages). The relative abundances of Hyphomicrobiales and Burkholderiales in the karst region were lower than those in the non-karst region (Figure 3a). With an increase in planting years, the relative abundance of Hyphomicrobiales in both the karst and non-karst regions increased.

At the genus level (>5%), *Methylobacterium* was dominant (12.44~29.66% in five stages) in the citrus soils from the karst region (Figure 3b), followed by *Beijerinckia* (9.12~19.68% in all five stages), *Pseudolabrys* (7.50~11.94% in all five stages), and *Bacillus* (5.04~8.50% in all five stages). *Bradyrhizobium* was dominant (32.79~42.94% in five stages) in the citrus soils from the non-karst region, followed by *Methylobacterium* (3.90~14.62% in all five stages). The relative abundance of *Methylobacterium* after the citrus cultivation in the karst region was lower than those in the NF and AL, while the relative abundance of *Bradyrhizobium* after the citrus cultivation in the non-karst region was higher than those in the NF and AL.

**Figure 4:**
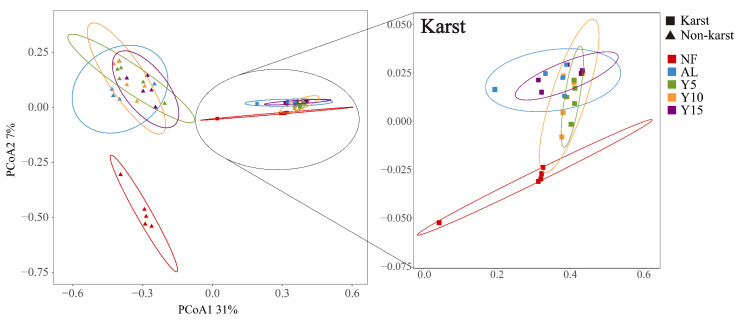
PCoA showed *phoD*-harboring bacterial community differed at different planting ages of citrus orchards in karst and non-karst regions. NF, natural forest; AL, abandoned land; Y5, 5-years planting; Y10, 10-years planting; Y15, 15-years planting.

The PCoA results reveal notable disparities in the *phoD*-harboring bacterial communities within the soil of the citrus orchards located in the karst and non-karst regions (Figure 4). The *phoD*-harboring bacterial community structures in the NF soil in the karst and non-karst regions were significantly different across different planting years and when compared with that in the AL, with the differences being particularly pronounced in the non-karst region.

**Figure 5:**
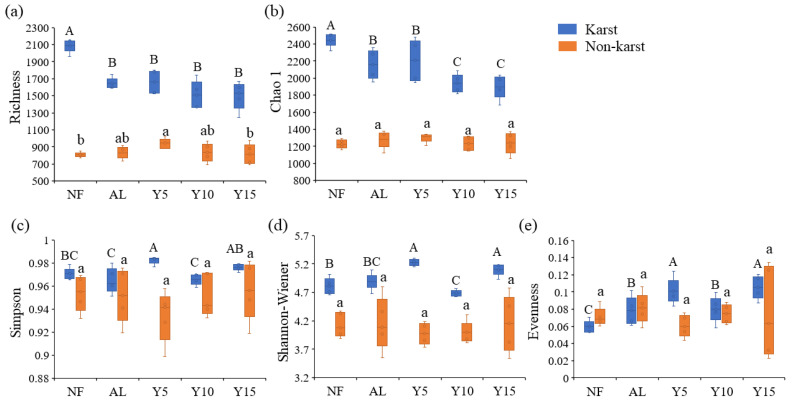
The Richness (**a**), Chao 1 (**b**), Simpson (**c**), Shannon-Wiener (**d**), and Evenness (**e**) indices of the *phoD*-harboring bacteria in different planting ages of the citrus orchards in the karst and non-karst regions. Different capital and lowercase letters indicate significant differences between the different planting ages in the karst and non-karst regions (*p* < 0.05), respectively. NF, natural forest; AL, abandoned land; Y5, 5-years planting; Y10, 10-years planting; Y15, 15-years planting.

The diversity indices (Richness, Chao 1, Simpson, and Shannon–Wiener indices) of the *phoD*-harboring bacteria in the soil of citrus orchards in the karst region were significantly higher than those in the non-karst region (Figure 5). In the karst region, the Richness and Chao 1 indices of the *phoD*-harboring bacteria in the citrus orchard soil were lower than those in the NF; however, the evenness indices were higher than those of the NF and AL. Conversely, in the non-karst region, the Richness and Chao 1 indices of the cultivated land exceeded those of the NF, and the Evenness indices were lower than those of the AL. The *phoD*-harboring microorganic Richness and Chao 1 indices of citrus soil in the karst region decreased with an increase in the planting years, whereas the Shannon–Wiener, Simpson, and Evenness indices increased after decreasing. The Simpson, Shannon–Wiener, and Evenness indices in the citrus soil of the non-karst region increased with an increase in the planting years.

### 3.3. Co-Occurrence Networks of phoD-Harboring Bacteria

**Figure 6:**
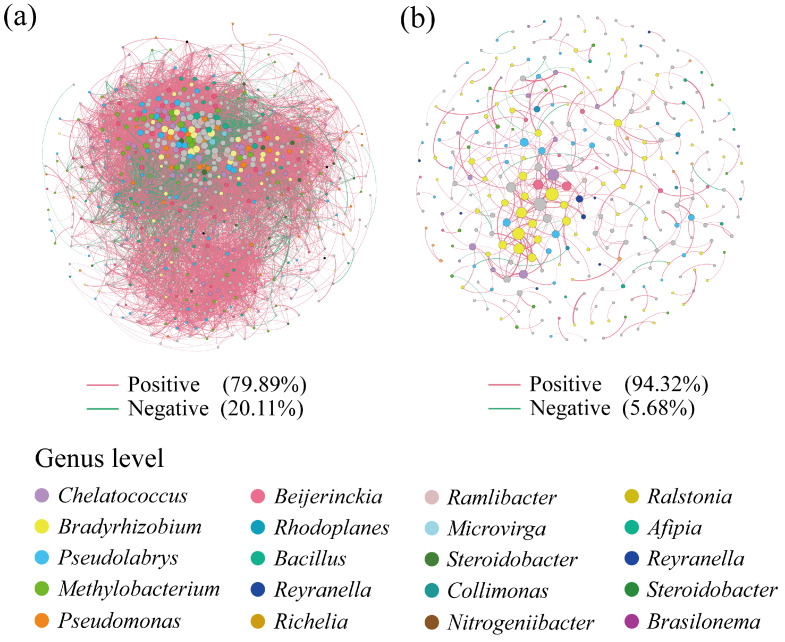
Co-occurrence networks of the soil *phoD*-harboring bacterial community at the genus level in the karst (**a**) and non-karst (**b**) regions. A red line indicates a positive relation, and a green line indicates a negative relation.

The co-occurrence networks of the soil *phoD*-harboring bacteria had 679 nodes and 18,618 edges within the karst citrus orchards and 315 nodes and 405 edges in the non-karst regions (Figure 6, Table S2). The average degree, clustering coefficient, and network densities of the soil co-occurrence network in the karst citrus orchards were significantly higher than those of the networks in the non-karst region, with 21.33-, 2.20-, and 10.13-fold higher values, respectively. Furthermore, in the Zi–Pi diagram, the soil co-occurrence network of citrus orchards in the karst region had 4.4-fold more key nodes than that in the non-karst region (Figure S1). *Chelatococcus* (10.67%), *Methylobacterium* (9.48%), *Pseudolabrys* (8.44%), *Bradyrhizobium* (7.7%), *Pseudomonas* (6.81%), and *Beijerinckia* (6.37%) were keystone genera in the co-occurrence networks of the *phoD*-harboring bacteria in the karst region citrus orchard soils. Conversely, in the non-karst region citrus orchards, *Bradyrhizobium* (26.35%) exhibited the most significant correlations with the other groups, followed by *Pseudolabrys* (10.16%), *Chelatococcus* (6.03%), and *Methylobacterium* (5.4%).

### 3.4. Soil phoD-Harboring Bacteria Affected P Availability

**Figure 7:**
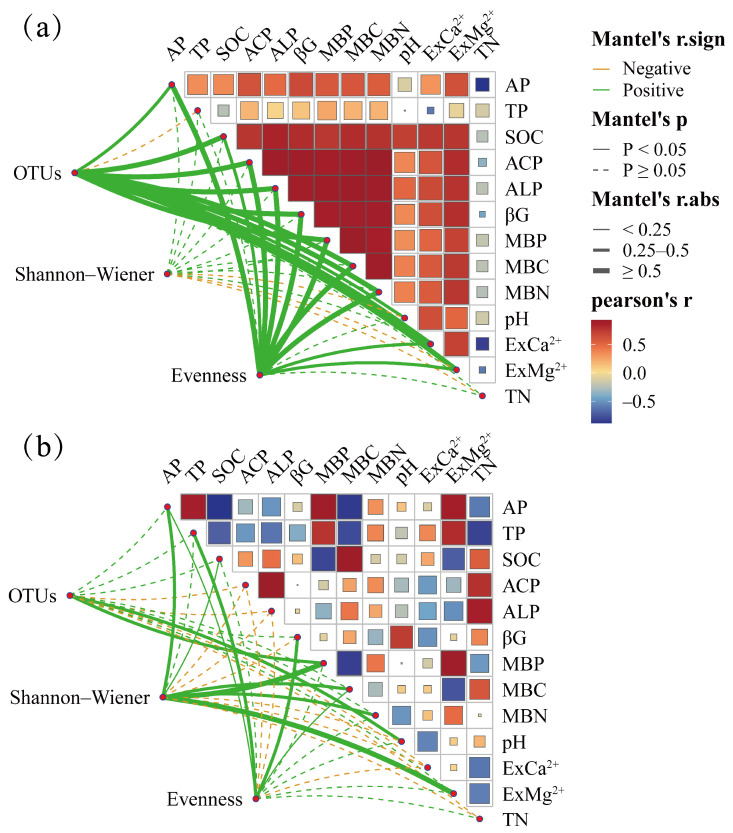
Relationships between the relative abundance of bacterial communities and the soil physicochemical parameters in the karst (**a**) and non-karst (**b**) ecosystems using the Mantel test.

According to the Mantel test results, the soil *phoD*-harboring microbial Richness, Chao 1, and Evenness indices were positively correlated with the soil AP and SOC content and phosphatase activity (ALP and ACP) in the citrus orchards in the karst region (Figure 7a). In the non-karst region, soil *phoD*-harboring microbial Evenness indices were significantly and positively correlated with the AP, TP, and MBN contents (Figure 7b). The AP in the karst region of the citrus orchards was positively correlated with the TP, SOC, ExMg^2+^, ExCa^2+^, MBP, MBN, and MBC contents, as well as the phosphatase activities (ALP and ACP), but negatively correlated with the TN content. In the non-karst region citrus orchards, the AP was significantly positively correlated with the TP, ExMg^2+^, and MBP contents, but negatively correlated with the SOC, MBC, and TN contents and ALP activity.

**Figure 8:**
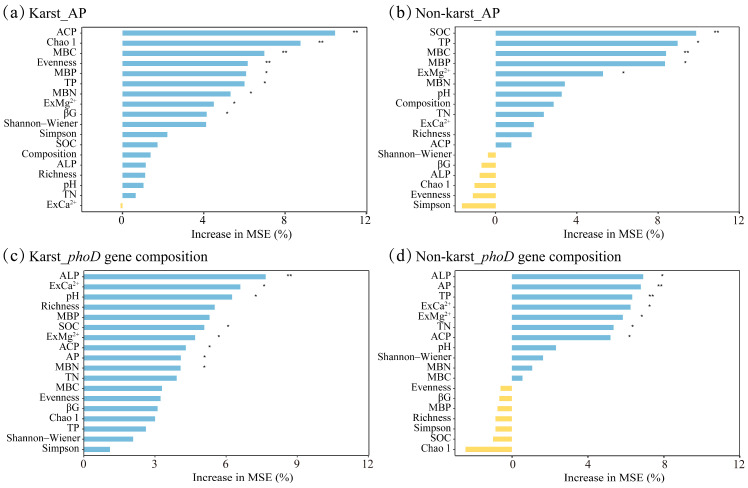
Ranking of important factors affecting available phosphorus (**a**,**b**) and *phoD*-harboring bacteria composition (**c**,**d**) in karst (**a**,**c**) and non-karst (**b**,**d**). ** *p* < 0.01; * *p* < 0.05.

The Random Forest modeling results revealed several factors as significant predictors of the AP variability within the karst region citrus orchards, including the ACP, Chao1 index, MBC, Evenness index, MBP, TP, MBN, ExMg^2+^, and βG (Figure 8a). In contrast, in the non-karst region citrus orchards, the SOC, TP, MBC, MBP, and ExMg^2+^ were the predictive factors for the AP content variability (Figure 8b). When examining the structural changes in the *phoD*-harboring bacteria within the karst region citrus orchards, the ALP, ExCa^2+^, pH, SOC, ExMg^2+^, ACP, AP, and MBN were crucial predictors (Figure 8c). Similarly, in the non-karst region citrus orchards, the ALP, AP, TP, ExCa^2+^, ExMg^2+^, TN, and ACP were significant predictors of the *phoD*-harboring bacterial community structure (Figure 8d).

**Figure 9:**
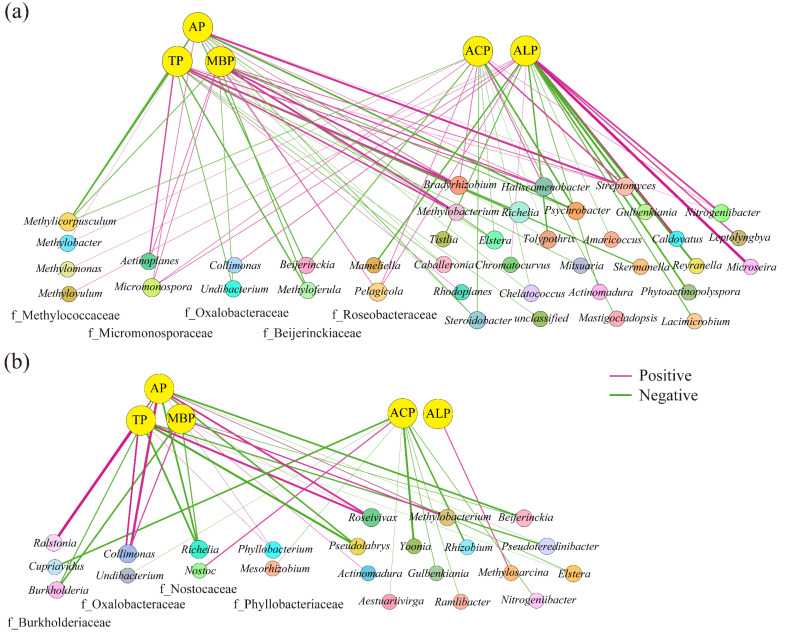
Correlation network between the *phoD*-harboring bacteria at the genus level and the soil P fractions and phosphatase activities. A green edge represents a negative interaction between two nodes, while a red edge represents a positive interaction between two nodes. (**a**) Karst; (**b**) non-karst.

Within the karst region, certain genera, including *Pelagicola*, *Methylobacter*, *Streptomyces*, and *Micromonospora*, displayed positive correlations with the AP, whereas other genera, such as *Beijerinckia*, *Chelatococcus*, *Psychrobacter*, *Elstera*, and *Methylicorpusculum*, exhibited negative correlations (Figure 9a). In contrast, in the non-karst region, the AP was positively correlated with genera such as *Roseivivax*, *Collimonas*, *Methylobacterium*, *Ralstonia*, and *Phyllobacterium*, whereas genera such as *Beijerinckia*, *Pseudolabrys*, *Burkholderia*, *Elstera*, *Richelia*, and *Pseudoteredinibacter* were negatively correlated with the AP (Figure 9b). These genera were not predominant among the *phoD*-harboring bacteria but were rather rare.

**Figure 10:**
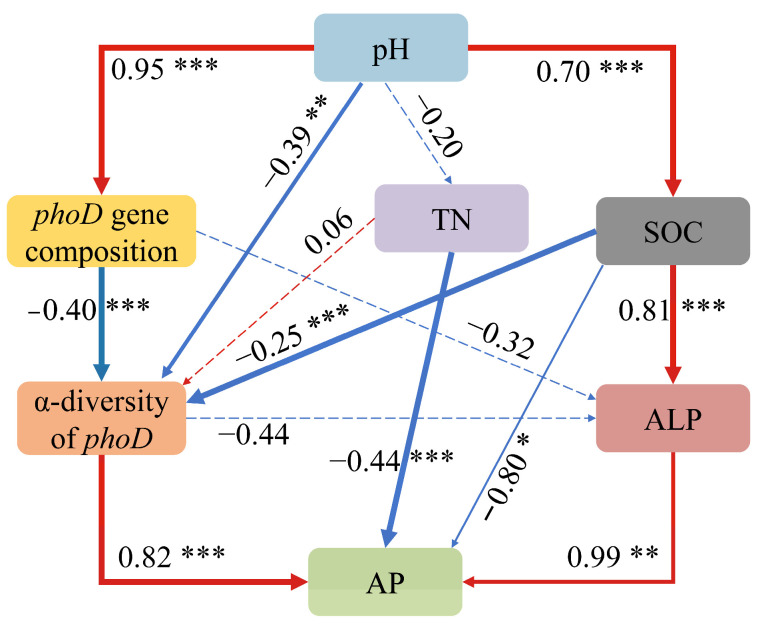
The relative abundance of *phoD*-harboring bacteria at order (**a**) and genus (**b**) levels in citrus orchards of different ages within karst and non-karst regions. *** *p* < 0.001; ** *p* < 0.01; * *p* < 0.05.

According to the SEM results, the pH and SOC were key environmental factors that affected the AP, and the citrus planting altered the soil pH, SOC, and total N, as well as the soil *phoD*-harboring bacterial community structure and diversity, which led to changes in the ALP activity, and, in turn, affected the P availability (Figure 10).

## 4. Discussion

### 4.1. Soil P Availability and Influencing Factors

In the present study, the AP contents of citrus planting soils were lower in the karst regions than in the non-karst regions, while those of the natural forest exhibited the opposite trends. The phenomena could be attributed to variations in the soil nutrient conditions. The soil TP content in the natural forest was higher in the karst region than in the non-karst region, which served as a significant source of AP. Additionally, a high Ca content and pH can facilitate Ca-P formation and enhance the P stability [32]. Notably, the soil pH values were significantly higher (6.64) in the natural forest land of the karst regions than in the non-karst regions (4.49), in addition to the higher ExCa^2+^ contents. The findings indicate that the soil P was more prone to Ca-P formation and the other P-binding forms in the karst regions, which resulted in a greater P stability when compared with that in non-karst regions.

The AP content decreased with an increase in the planting duration in the karst regions, whereas it increased in the non-karst regions. The results are consistent with the findings of previous studies that showed that the soil AP content decreased from 2.59 mg kg^−1^ after 5 years of planting to 1.20 mg kg^−1^ after 10 years of planting in karst regions [33], while it increased from 17.47 mg kg^−1^ to 26.92 mg kg^−1^ over 1–15 years in non-karst regions [34]. This observation may be related to the impact of crop cultivation on biochemical reactions. Notably, soil microbes, secreting phosphatase and accumulating P, play key roles in P transformation [35]. In the present study, citrus cultivation reduced the soil ALP and ACP activities and the MBP content when compared with the levels in the natural forest in the karst regions. The results indicate that the amounts of microbes that participated in the P accumulation decreased after the citrus cultivation, with corresponding reductions in phosphatase. This might lead to a decrease in the soil AP after the citrus cultivation in the karst regions. Although the soil ALP and ACP activities also decreased after the citrus cultivation in the non-karst regions, the MBP contents of the cultivated soils increased dramatically, where they exhibited values that were approximately 5.5-, 14.4-, and 29.1-fold greater after 5, 10, and 15 years of planting, respectively. In general, MBP is considered as a type of soil available P. Thus, increasing soil AP after citrus cultivation in karst regions may be primarily related to the increasing MBP amounts. The above results highlight the potential importance of soil microbes in soil AP enhancement, which also supports our first hypothesis.

### 4.2. phoD-Harboring Microbial Community and Co-Occurrence Networks and Influencing Factors

The relative abundance of *Methylobacterium*, a dominant genus in the citrus soils of the karst regions, was lower than that in the natural forest and abandoned land, with an initial decrease followed by an increase, with an increase in the planting years. This observation may be related to the impacts of crop cultivation on the biomass. Methylotrophic microbes associated with crops could enhance plant growth by increasing the biomass, N content, and yield [36]. In the present study, the citrus cultivation reduced the soil TP and MBP contents when compared with the levels in the natural forest and abandoned land, with an initial decrease followed by an increase, with an increase in the planting years in the karst regions. The results indicate that the number of microbes that participated in the P accumulation varied according to the *Methylobacterium* abundance following the citrus cultivation. In the non-karst citrus soils, however, the dominant genus *Bradyrhizobium* had a higher relative abundance than that in the natural forests and abandoned lands, and its relative abundance increased with the extension of the planting years. The genus *Bradyrhizobium*, a symbiotic N-fixing microorganism [37], plays a pivotal role in the N cycle [22]. Specifically, it is instrumental in the biological fixation process and subsequent mineralization, both of which can promote the conversion of inactive P into its active form [38,39].

The *phoD*-harboring bacterial community structure was significantly different between the karst and non-karst regions, with marked differences between the different planting years in the non-karst regions. This observation may be related to geological conditions and differences in the soil nutrients. Each species is adapted to specific physical and chemical conditions, and its abundance is constrained by the environmental factors affecting growth [40]. The karst regions demonstrated elevated levels of SOC, ExCa^2+^, and ExMg^2+^ compared with their non-karst counterparts. The soil nutrient (SOC, ExCa^2+^, and ExMg^2+^) contents increased significantly following the citrus planting in the non-karst regions; however, the soil nutrient contents were still higher in the karst regions than in the non-karst regions. The favorable soil conditions provided a dynamic ecological environment for microorganisms, which was conducive for bound P formation. In the present study, the relative abundances of *Methylobacterium*, *Beijerinckia*, and *Pseudolabrys* were higher in the karst regions, and the percentages of *Bradyrhizobium* were higher in the non-karst regions. As a result, the bacteria that harbored *phoD* in the karst region soil were not only more abundant but also occupied a more significant position in the overall microbial community in the karst region. In such an environment, microbial interactions are likely to establish a comparatively stable ecological equilibrium, resulting in minimal differentiation within the microbial communities [41]. In addition, differences in soil nutrients existed in the non-karst regions with varying planting years. This variation led to dynamic changes in the abundance of the *phoD*-harboring dominant bacteria in the soil of different planting years, which significantly impacted the soil P cycle and plant P absorption [42], and thereby exacerbated the differentiation within the microbial community.

In the present study, the diversity indices and network complexity of the *phoD*-harboring bacteria were higher in the karst regions compared with the non-karst regions. This was related to the unique geological and environmental conditions in the karst regions, such as the complex underground water systems, high humidity, and abundant organic matter. Simultaneously, the karst ecosystem exhibits a significant degree of heterogeneity [43], and the high soil nutrient content results in higher microbial diversity compared with non-karst regions. In addition, the results are consistent with previous studies that demonstrated that the greater complexity of the microbial network is associated with increased diversity [44,45]. In comparison with non-karst regions, the high diversity of *phoD*-harboring bacteria in karst citrus orchards promotes interspecific interactions, contributing to the establishment of complex microbial networks. 

### 4.3. Mechanism by Which phoD-Harboring Bacteria Influenced P Availability

In the present study, the rare genera of soil *phoD*-harboring bacteria were associated with the increased AP in both the karst and non-karst regions. Several plausible explanations exist for the observation. First, unique geological and environmental conditions in different regions could have facilitated the adaptation and survival of rare genera. Such bacteria can promote organic phosphate mineralization by secreting ALP to meet their P requirements [46]. *Beijerinckia* and *Streptomyces* represent such bacteria [38,47]. Second, non-abundant and rare species are critical for microbial community stability. According to the findings of the present study, 91% of the keystone nodes in the karst region and 60% of those in the non-karst region were associated with non-dominant and rare genera. The species contribute to soil microbial community stability through their specialized metabolic functions [48]. The results further support the notion that sub-abundant and rare *phoD*-harboring bacteria species are more critical in P-transformation processes within citrus orchards in both karst and non-karst regions than their dominant counterparts.

Under distinct karst and non-karst geological conditions, citrus planting has significant effects on soil pH, SOC, and TN. In the present study, the soil pH and SOC contents influenced the compositions of the microbial communities, consistent with previous studies [49,50]. The strong correlation between the soil pH and bacterial community structure can be attributed to the narrow growth tolerances exhibited by most microbes and the increasingly challenging conditions in karst environments [51]. SOC plays a pivotal role in microbial growth and reproduction, with the majority of microbes exhibiting marked and positive correlations with SOC content [40]. It also impacts species interactions, which, in turn, participate in the regulation of soil nutrient cycling through non-dominant or rare genera [45]. In the present study, the soil pH and SOC contents significantly impacted the *phoD*-harboring bacterial community diversity through the bacterial community structure, and thereby influenced the AP in soil. This is ascribed to the variations in the availability of soil nutrients and the alterations in the microbial living environment. Most *phoD*-harboring bacteria have a narrow tolerance for growth conditions. As a result, they are subject to intense competitive selection, leading to alterations in the soil bacterial community structure and diversity. Changes in the soil bacterial community diversity imply that the survival and metabolic activities of soil bacterial taxa, especially rare taxa and keystone taxa, are impacted, which might lead to variations in the soil organic phosphate mineralization efficiency and subsequently affect the change in the soil AP content [16]. For instance, *Streptomyces*, a rare bacterium in the karst regions, was positively correlated with the content of AP. This rare bacterium can convert insoluble phosphorus into available phosphorus via acid hydrolysis and enzymatic hydrolysis, thereby raising the content of AP. Similarly, *Methylobacterium*, a rare bacterium in the non-karst regions, was positively correlated with the content of AP in the soil. This rare bacterium can markedly enhance the ALP activity of the soil, thereby facilitating the mineralization of organophosphorus and increasing the content of AP in the soil [16]. Thus, the alteration of the microbial community structure had an impact on the ALP activity, which subsequently influenced the organophosphorus decomposition in the soil, and thereby affected the P utilization efficiency of the citrus. Therefore, adjusting nutrient management practices and regulating the *phoD* of non-dominant or rare species is crucial for managing soil AP in citrus orchards, both in karst and non-karst regions.

## 5. Conclusions

The present study provides insights into how *phoD*-harboring bacteria regulate soil available P in karst and non-karst citrus orchard soils across a planting duration gradient. The soil AP contents in the karst soils were lower than those in the non-karst soils following prolonged citrus planting. The AP decreased in the karst soils but increased in the non-karst soils with an increase in the citrus planting duration. However, the diversity indices and network complexity of the *phoD*-harboring bacteria were higher in the karst regions than in the non-karst regions. Additionally, there were marked community differences between the microbes across different planting years in the non-karst regions. Notably, the rare *phoD*-harboring bacterial genera were positively correlated with the AP in the two lithological soils. The SEM results demonstrated that the pH and SOC were key environmental factors that influenced the AP. Furthermore, the citrus cultivation altered both the pH and SOC, which consequently affected the *phoD*-harboring bacterial community structure, along with their co-occurrence network connectivity and diversity, which ultimately impacted the ALP secretion and AP. The findings of the present study suggest that the rare *phoD*-harboring bacterial genera played a more significant role than the dominant genera in enhancing the AP in both the karst and non-karst regions, particularly with an increased duration of citrus planting. However, most *phoD*-harboring bacteria with a high P conversion capacity were uncultured. Thus, future studies should attempt to identify bacterial strains that exhibit exceptional proficiency in P transformation, with an emphasis on their potential for extensive application in agricultural practices and ecological restoration projects within karst regions.

## Data Availability

The raw data supporting the conclusions of this article will be made available by the authors on request.

## Supplementary Materials

The following supporting information can be downloaded from https://www.mdpi.com/article/10.3390/microorganisms12122582/s1—Figure S1: The relative abundance of *phoD*-harboring bacteria at the phylum level in different age of citrus orchards in the karst and non-karst regions. NF, natural forest soil; AL, abandoned land soil; Y5, 5-year citrus soil; Y10, 10-year citrus soil; Y15, 15-year citrus soil. Figure S2: The within-modules connectivity (Zi) and between-modules connectivity (Pi) (Zi-Pi) plot display keystone species of *phoD* community in the treatments of the karst (a) and non-karst (b). Module hubs were identified as Zi > 2.5, Pi < 0.62; connectors were identified as Zi < 2.5, Pi > 0.62; network hubs were identified as Zi ≥ 2.5, Pi > 0.62. Figure S3: Co-occurrence networks of the soil *phoD*-harboring bacterial community at the phylum level in the karst (a) and non-karst (b) regions. A red line indicates a positive relation, and a green line indicates a negative relation. Figure S4: Correlation network between the *phoD*-harboring bacteria at the phylum level and the soil P fractions and phosphatase activities. A green edge represents a negative interaction between two nodes, and a red edge represents a positive interaction between two nodes. (a) Karst; (b) non-karst. Figure S5: Relationship between the relative abundance of *phoD*-harboring bacteria at the genus level (top 19) and physicochemical parameters in the karst (a) and non-karst (b), as well as relationships between the microbial keystone taxa and physicochemical parameters in the karst (c) and non-karst (d). ** *p* < 0.01; * *p* < 0.05. Table S1: Soil physical and chemical properties in different ages of citrus orchards with karst and non-karst. Table S2: Soil microbial co-occurrence network structure properties under different landforms.

## References

[1] Zhu F., Qu L., Hong X., Sun X. (2011). Isolation and Characterization of a Phosphate-Solubilizing Halophilic Bacterium Kushneria sp. YCWA18 from Daqiao Saltern on the Coast of Yellow Sea of China. Evid. Based Complement. Altern. Med..

[2] Ma X., Li F., Chen Y., Chang Y., Lian X., Li Y., Ye L., Yin T., Lu X. (2022). Effects of Fertilization Approaches on Plant Development and Fertilizer Use of Citrus. Plants.

[3] Li Y.-J., Yang M., Zhang Z.-Z., Li W.-L., Guo C.-Y., Chen X.-P., Shi X.-J., Zhou P., Tang X.-D., Zhang Y.-Q. (2019). An Ecological Research on Potential for Zero-growth of Chemical Fertilizer Use in Citrus Production in China. Ekoloji.

[4] White P.J., Brown P.H. (2010). Plant nutrition for sustainable development and global health. Ann. Bot..

[5] Zancanaro L.E., Nunes R., Sousa D., Busato J., Figueiredo C. (2019). Response of Maize to Different Soil Residual Phosphorus Conditions. Agron. J..

[6] Carpenter S. (2005). Eutrophication of Aquatic Ecosystems: Bistability and Soil Phosphorus. Proc. Natl. Acad. Sci. USA.

[7] Chen X., Condron L.M., Dunfield K.E., Wakelin S.A., Chen L. (2021). Impact of grassland afforestation with contrasting tree species on soil phosphorus fractions and alkaline phosphatase gene communities. Soil Biol. Biochem..

[8] Zeng Q., Mei T., Delgado-Baquerizo M., Wang M., Tan W. (2022). Suppressed phosphorus-mineralizing bacteria after three decades of fertilization. Agric. Ecosyst. Environ..

[9] Bibi S., Irshad M., Ullah F., Mahmood Q., Shahzad M., Tariq M.A.U.R., Hussain Z., Mohiuddin M., An P., Ng A.W.M. (2023). Phosphorus extractability in relation to soil properties in different fields of fruit orchards under similar ecological conditions of Pakistan. Front. Ecol. Evol..

[10] MacDonald G.K., Bennett E.M., Taranu Z.E. (2012). The influence of time, soil characteristics, and land-use history on soil phosphorus legacies: A global meta-analysis. Glob. Change Biol..

[11] Jiang Y., Yang X., Ni K., Ma L., Shi Y., Wang Y., Cai Y., Ma Q., Ruan J. (2023). Nitrogen addition reduces phosphorus availability and induces a shift in soil phosphorus cycling microbial community in a tea (*Camellia sinensis* L.) plantation. J. Environ. Manag..

[12] Fraser T.D., Lynch D.H., Bent E., Entz M.H., Dunfield K.E. (2015). Soil bacterial *phoD* gene abundance and expression in response to applied phosphorus and long-term management. Soil Biol. Biochem..

[13] Tan H., Barret M., Mooij M.J., Rice O., Morrissey J.P., Dobson A., Griffiths B., O’Gara F. (2013). Long-term phosphorus fertilisation increased the diversity of the total bacterial community and the *phoD* phosphorus mineraliser group in pasture soils. Biol. Fertil. Soils.

[14] Daneshgar S., Callegari A., Capodaglio A.G., Vaccari D. (2018). The Potential Phosphorus Crisis: Resource Conservation and Possible Escape Technologies: A Review. Resources.

[15] Lynch M.D.J., Neufeld J.D. (2015). Ecology and exploration of the rare biosphere. Nat. Rev. Microbiol..

[16] Wei X., Hu Y., Razavi B.S., Zhou J., Shen J., Nannipieri P., Wu J., Ge T. (2019). Rare taxa of alkaline phosphomonoesterase-harboring microorganisms mediate soil phosphorus mineralization. Soil Biol. Biochem..

[17] Tong X., Brandt M., Yue Y., Horion S., Wang K., Keersmaecker W.D., Tian F., Schurgers G., Xiao X., Luo Y. (2018). Increased vegetation growth and carbon stock in China karst via ecological engineering. Nat. Sustain..

[18] Wang Y.-P., Huang Y., Augusto L., Goll D.S., Helfenstein J., Hou E. (2022). Toward a Global Model for Soil Inorganic Phosphorus Dynamics: Dependence of Exchange Kinetics and Soil Bioavailability on Soil Physicochemical Properties. Glob. Biogeochem. Cycles.

[19] Green S.M., Dungait J.A.J., Tu C., Buss H.L., Sanderson N., Hawkes S.J., Xing K., Yue F., Hussey V.L., Peng J. (2019). Soil functions and ecosystem services research in the Chinese karst Critical Zone. Chem. Geol..

[20] Zhou M., Yang H., Zhu T., Zhang C., Zhu D. (2022). Preliminary Research on Agricultural Cultivation Decreasing Amino Sugar Accumulation in Calcareous Soils in Subtropical Karst Region of China. Land.

[21] Sun M., Yang R., Tang Y., Xiao D., Zhang W., Xu Z., Shi Z., Hu P., Wu H., Wang K. (2023). Lithologic control of soil C:N:P stoichiometry across a climatic gradient in southwest China. J. Soil Sediment..

[22] Pan F., Yu X., Chen M., Liang Y. (2024). Vegetation recovery reshapes the composition and enhances the network connectivity of phoD-harboring microorganisms to promote P availability in a karst ecosystem. Sci. Total Environ..

[23] Finzi A.C., Sinsabaugh R.L., Long T.M., Osgood M.P. (2006). Microbial Community Responses to Atmospheric Carbon Dioxide Enrichment in a Warm-Temperate Forest. Ecosystems.

[24] Sakurai M., Wasaki J., Tomizawa Y., Shinano T., Osaki M. (2008). Analysis of bacterial communities on alkaline phosphatase genes in soil supplied with organic matter. Soil Sci. Plant Nutr..

[25] Caporaso J.G., Kuczynski J., Stombaugh J., Bittinger K., Bushman F.D., Costello E.K., Fierer N., Peña A.G., Goodrich J.K., Gordon J.I. (2010). QIIME allows analysis of high-throughput community sequencing data. Nat. Methods.

[26] Fish J.A., Chai B., Wang Q., Sun Y., Brown C.T., Tiedje J.M., Cole J.R. (2013). FunGene: The functional gene pipeline and repository. Front. Microbiol..

[27] Faust K. (2021). Open challenges for microbial network construction and analysis. ISME J..

[28] Berry D., Widder S. (2014). Deciphering microbial interactions and detecting keystone species with co-occurrence networks. Front. Microbiol..

[29] Sun Y., Li X., Cao N., Duan C., Ding C., Huang Y., Wang J. (2022). Biodegradable microplastics enhance soil microbial network complexity and ecological stochasticity. J. Hazard. Mater..

[30] Guimerà R., Nunes Amaral L.A. (2005). Functional cartography of complex metabolic networks. Nature.

[31] Xiao X., Fan M., Wang E., Chen W., Wei G. (2017). Interactions of plant growth-promoting rhizobacteria and soil factors in two leguminous plants. Appl. Microbiol. Biotechnol..

[32] Hinsinger P. (2001). Bioavailability of soil inorganic P in the rhizosphere as affected by root-induced chemical changes: A review. Plant Soil.

[33] Yang R., Li J., Long J., Liao H., Wang X., Li Y. (2021). Structural characteristics of bacterial community in rhizosphere soil of Zanthoxylum bungeamun in different planting years in Karst Areas of Guizhou. Ecol. Environ. Sci..

[34] Liao L., Shi F., Zhang N., Chen X., Bu H., Sun F. (2022). Effects of Different Planting Years on Rhizosphere Soil Physiochemical Properties and Microbial Community of *Zanthoxylum bungeanum*. Bull. Bot. Res..

[35] Peng S., Kuang X., Cheng H., Wei K., Cai K., Tian J. (2024). Post-agricultural succession affects the accumulation and enzymatic transformation of organic phosphorus in a karst area, southwest China. Plant Soil.

[36] Kumar M., Kour D., Yadav A.N., Saxena R., Rai P.K., Jyoti A., Tomar R.S. (2019). Biodiversity of methylotrophic microbial communities and their potential role in mitigation of abiotic stresses in plants. Biologia.

[37] Wielbo J., Kidaj D., Koper P., Kubik-Komar A., Skorupska A. (2012). The effect of biotic and physical factors on the competitive ability of Rhizobium leguminosarum. Cent. Eur. J. Biol..

[38] Luo G., Ling N., Nannipieri P., Chen H., Raza W., Wang M., Guo S., Shen Q. (2017). Long-term fertilisation regimes affect the composition of the alkaline phosphomonoesterase encoding microbial community of a vertisol and its derivative soil fractions. Biol. Fertil. Soils.

[39] Zhu X., Zhao X., Lin Q., Li G. (2021). Distribution Characteristics of *phoD*-Harbouring Bacterial Community Structure and Its Roles in Phosphorus Transformation in Steppe Soils in Northern China. J. Soil Sci. Plant Nutr..

[40] Fan Z., Lu S., Liu S., Guo H., Wang T., Zhou J., Peng X. (2019). Changes in Plant Rhizosphere Microbial Communities under Different Vegetation Restoration Patterns in Karst and Non-karst Ecosystems. Sci. Rep..

[41] Barber J.N., Nicholson L.C., Woods L.C., Judd L.M., Sezmis A.L., Hawkey J., Holt K.E., McDonald M.J. (2022). Species interactions constrain adaptation and preserve ecological stability in an experimental microbial community. ISME J..

[42] Wang L., Wang J., Yuan J., Tang Z., Wang J., Zhang Y. (2023). Long-Term Organic Fertilization Strengthens the Soil Phosphorus Cycle and Phosphorus Availability by Regulating the *pqqC*- and *phoD*-Harboring Bacterial Communities. Microb. Ecol..

[43] Xiao D., He X., Zhang W., Cheng M., Hu P., Wang K. (2022). Diazotroph and arbuscular mycorrhizal fungal diversity and community composition responses to karst and non-karst soils. Appl. Soil Ecol..

[44] Wagg C., Schlaeppi K., Banerjee S., Kuramae E.E., van der Heijden M.G.A. (2019). Fungal-bacterial diversity and microbiome complexity predict ecosystem functioning. Nat. Commun..

[45] Xiao D., He X., Zhang W., Hu P., Sun M., Wang K. (2022). Comparison of bacterial and fungal diversity and network connectivity in karst and non-karst forests in southwest China. Sci. Total Environ..

[46] Xu L., Cao H., Li C., Wang C., He N., Hu S., Yao M., Wang C., Wang J., Zhou S. (2022). The importance of rare versus abundant phoD-harboring subcommunities in driving soil alkaline phosphatase activity and available P content in Chinese steppe ecosystems. Soil Biol. Biochem..

[47] Olanrewaju O.S., Babalola O.O. (2019). Streptomyces: Implications and interactions in plant growth promotion. Appl. Microbiol. Biotechnol..

[48] Xun W., Liu Y., Li W., Ren Y., Xiong W., Xu Z., Zhang N., Miao Y., Shen Q., Zhang R. (2021). Specialized metabolic functions of keystone taxa sustain soil microbiome stability. Microbiome.

[49] Zhao S., Liu J.-J., Banerjee S., Zhou N., Zhao Z.-Y., Zhang K., Tian C.-Y. (2018). Soil pH is equally important as salinity in shaping bacterial communities in saline soils under halophytic vegetation. Sci. Rep..

[50] Li D., Chen L., Xu J., Ma L., Olk D.C., Zhao B., Zhang J., Xin X. (2018). Chemical nature of soil organic carbon under different long-term fertilization regimes is coupled with changes in the bacterial community composition in a Calcaric Fluvisol. Biol. Fertil. Soils.

[51] Pan F.J., Yang Q., Liang Y.M., Yu X., Hu P.L., Zhang W., Pang Y.L. (2024). Lithology and elevated temperature impact *phoD*-harboring bacteria on soil available P enhancing in subtropical forests. Sci. Total Environ..

