# Supplementary material for: Planted Citrus Regulates the Community and Networks of phoD-Harboring Bacteria to Drive Phosphorus Availability Between Karst and Non-Karst Soils
Source: Microorganisms. 2024 Dec 13;12(12):2582. doi: 10.3390/microorganisms12122582 (PMC11678004; doi:10.3390/microorganisms12122582)
Supplement: Supplementary file 1 [file microorganisms-12-02582-s001.zip › Supplementary Figures.pdf]

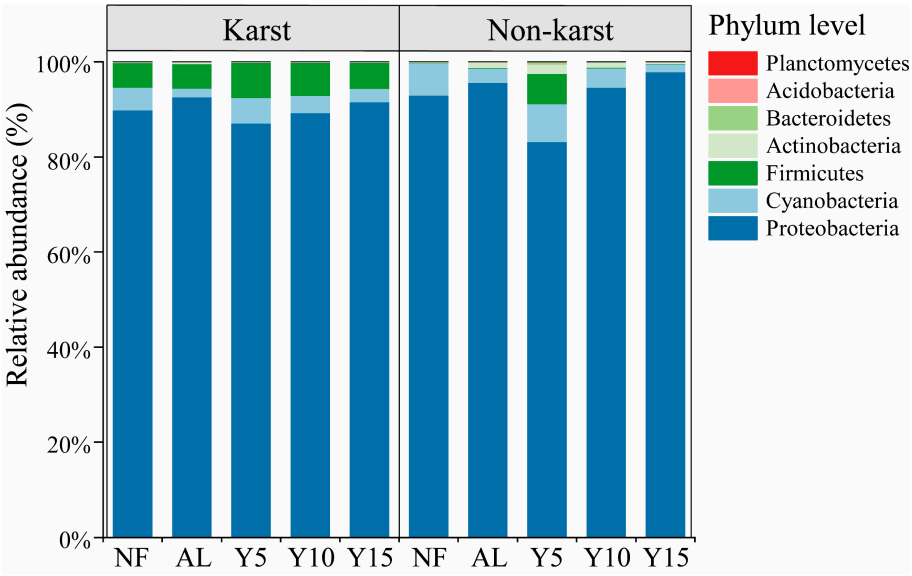

Figure S1 The relative abundance of *phoD*-harboring bacteria at the phylum level in different age of citrus orchards in the karst and non-karst regions. NF, natural forest soil; AL, abandoned land soil; Y5, 5-year citrus soil; Y10, 10-year citrus soil; Y15, 15-year citrus soil.

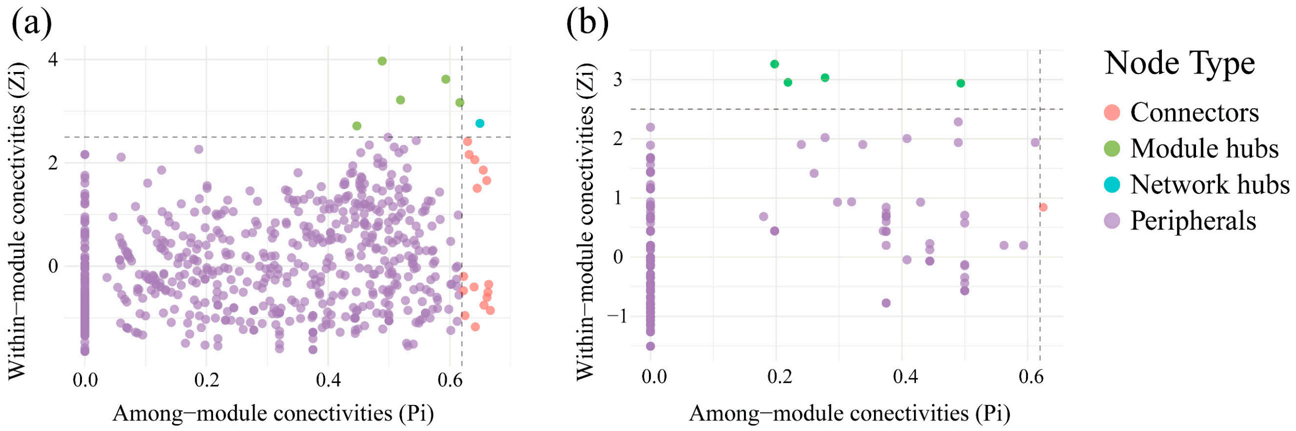

Figure S2 The within-modules connectivity (Zi) and between-modules connectivity (Pi) (Zi-Pi) plot display keystone species of *phoD* community in the treatments of the karst (a) and non-karst (b). Module hubs were identified as Zi > 2.5, Pi < 0.62; connectors were identified as Zi < 2.5, Pi > 0.62; network hubs were identified as Zi ⩾ 2.5, Pi > 0.62.

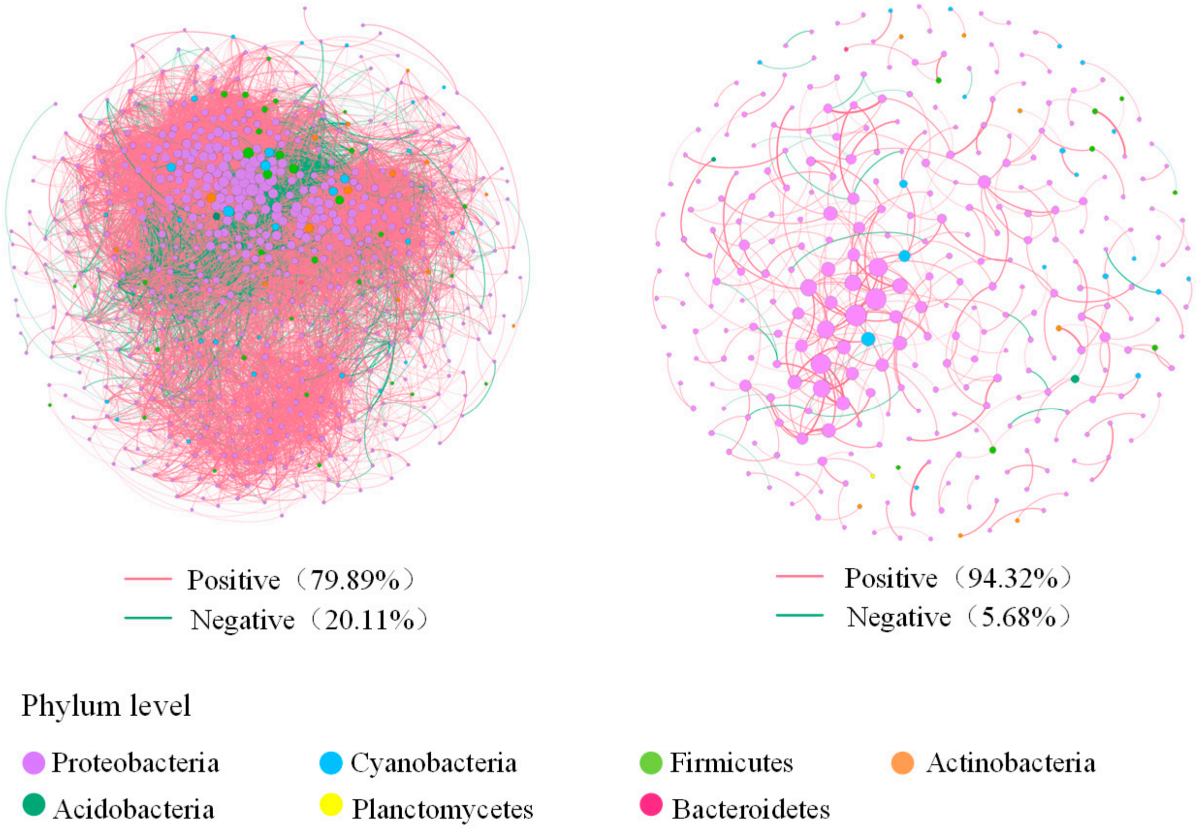

Figure S3 Co-occurrence networks of the soil *phoD*-harboring bacterial community at the phylum level in the karst (a) and non-karst (b) regions. A red line indicates a positive relation, and a green line indicates a negative relation.

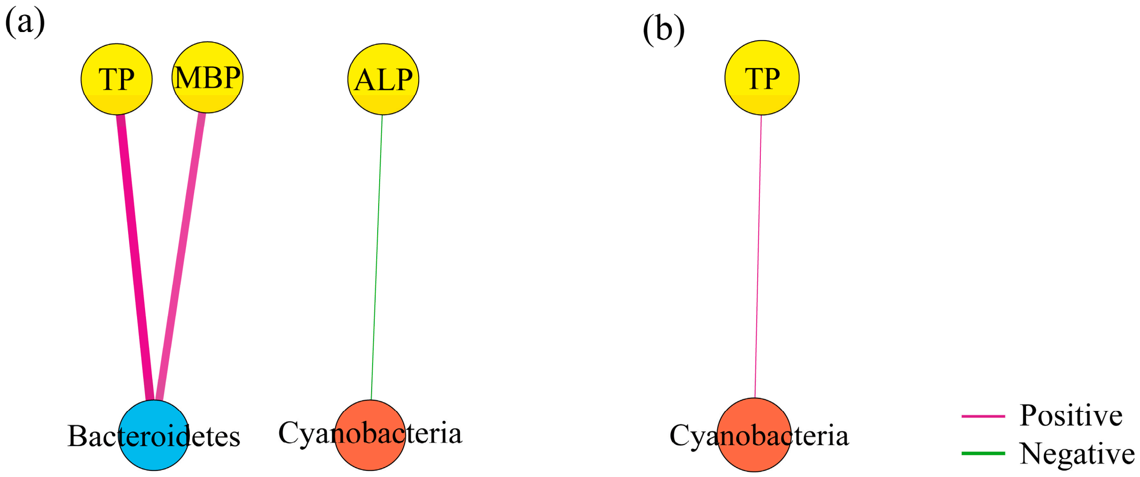

Figure S4 Correlation network between the *phoD*-harboring bacteria at the phylum level and the soil P fractions and phosphatase activities. A green edge represents a negative interaction between two nodes, and a red edge represents a positive interaction between two nodes. (a) Karst; (b) non-karst.

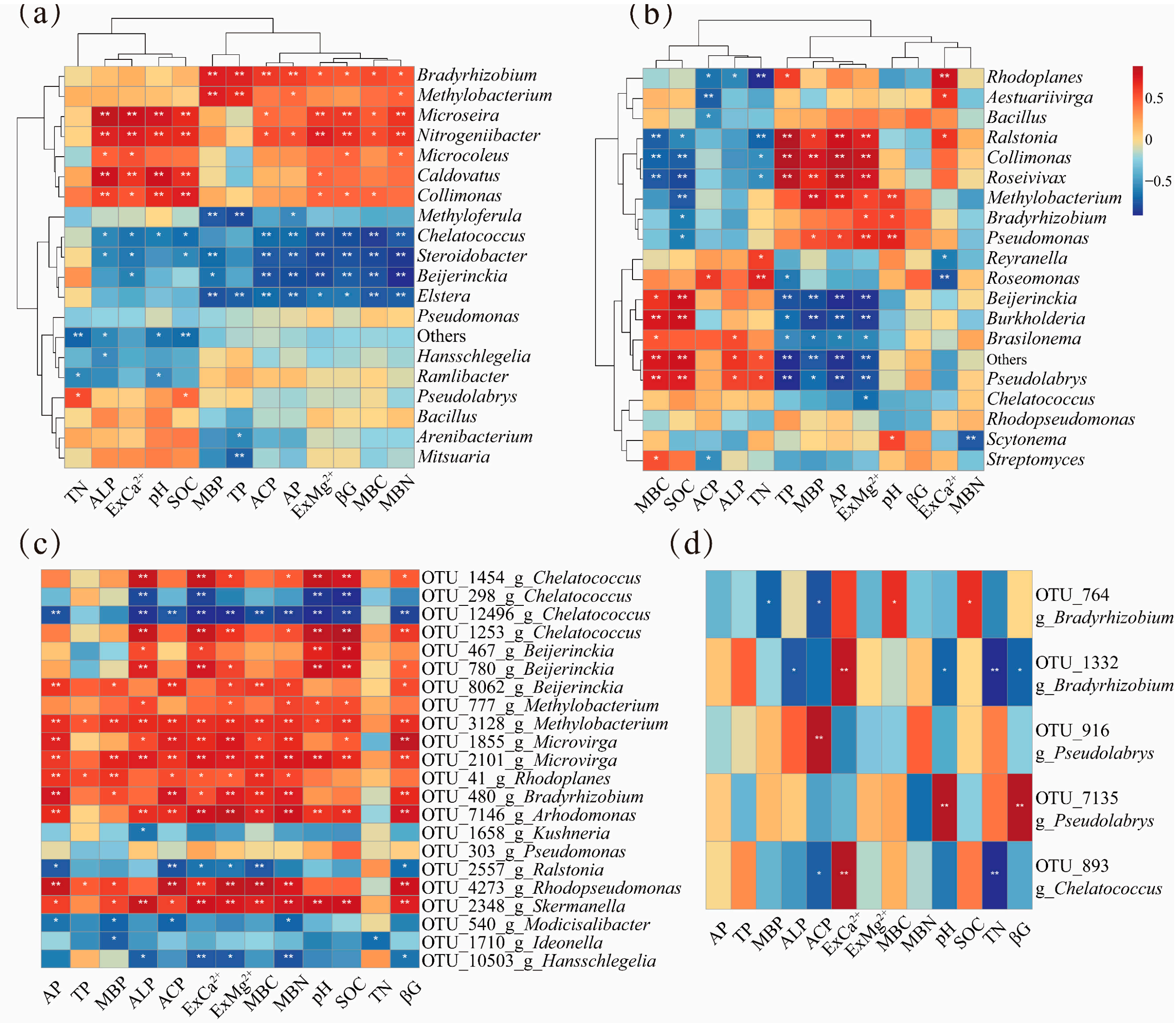

Figure S5 Relationship between the relative abundance of *phoD*-harboring bacteria at the genus level (top 19) and physicochemical parameters in the karst (a) and non-karst (b), as well as relationships between the microbial keystone taxa and physicochemical parameters in the karst (c) and non-karst (d). $^{**}p < 0.01$; $^{*}p < 0.05$.
