# Supplementary material for: Planted Citrus Regulates the Community and Networks of phoD-Harboring Bacteria to Drive Phosphorus Availability Between Karst and Non-Karst Soils
Source: Microorganisms. 2024 Dec 13;12(12):2582. doi: 10.3390/microorganisms12122582 (PMC11678004; doi:10.3390/microorganisms12122582)
Supplement: Supplementary file 1 [file microorganisms-12-02582-s001.zip › Supplementary Tables.pdf]

Table S1 Soil physical and chemical properties in different ages of citrus orchards with karst and non-karst.

landform	years	pH	$ExCa^{2+}$ mg • kg^{-1}	$ExMg^{2+}$ mg • kg^{-1}	MBC mg • kg^{-1}	MBN mg • kg^{-1}	βG nmol·h^{-1}·g^{-1}	TN %
karst	NF	6.64±0.02 Aa	219.75±4.61 Aa	1.855±0.148 Aa	3503.84±151.36 Aa	568.06±33.04 Aa	0.395±0.010 Aa	0.861±0.017 Bb
	AL	6.24±0.05 Ac	127.37±2.03 Ad	1.022±0.020 Ac	1049.31±41.57 Aabc	105.92±7.24 Aab	0.149±0.014 Abc	0.359±0.003 Ac
	Y5	6.56±0.05 Aa	184.58±1.78 Ab	1.331±0.053 Ab	1078.31±14.02 Aab	112.70±5.61 Aab	0.163±0.017 Ab	0.473±0.004 Bbc
	Y10	6.43±0.01 Ab	166.07±3.49 Ac	0.907±0.031 Ad	895.29±30.20 Ac	90.28±0.88 Ab	0.117±0.007 Acd	0.528±0.042 Abc
	Y15	6.41±0.01 Ab	107.57±6.22 Ae	0.816±0.050 Ae	895.29±24.24 Abc	74.44±2.11 Ac	0.095±0.007 Ad	1.264±0.124 Aa
Non-karst	NF	4.49±0.01 Bbc	0.00±0.00 Bc	0.055±0.002 Bc	1142.87±36.19 Ba	40.30±3.07 Bab	0.063±0.007 Bb	3.225±0.062 Aa
	AL	4.31±0.05 Bc	7.39±0.34 Ba	0.234±0.014 Bb	1100.55±23.50 Aa	36.58±4.29 Babc	0.050±0.004 Bb	0.161±0.001 Bc
	Y5	4.83±0.13 Ba	0.36±0.01 Bbc	0.362±0.033 Bab	1125.90±71.96 Aa	30.77±1.89 Bbc	0.086±0.004 Ba	1.714±0.035 Ab
	Y10	4.74±0.04 Bab	0.38±0.02 Bbc	0.390±0.031 Bab	745.00±34.85 Bb	29.11±1.34 Bc	0.059±0.006 Bb	0.331±0.006 Bbc
	Y15	4.53±0.05 Babc	2.36±0.29 Bb	0.875±0.107 Aa	700.04±51.79 Bb	49.91±0.81 Ba	0.058±0.005 Bb	0.298±0.022 Bbc

Different lowercase letters indicate significant differences between different citrus plantation ages; different capital letters indicate significant differences between different landform ($p < 0.05$). NF, natural forest soil; AL, abandoned land soil; Y5, 5-year citrus soil; Y10, 10-year citrus soil; Y15, 15-year citrus soil.

Table S2 Soil microbial co-occurrence network structure properties under different landforms.

network structure properties	*phoD*-harboring karst	non-karst
Average degree	54.839	2.571
average aggregation coefficient	0.503	0.229
average path length	2.636	6.868
network density	0.081	0.008
network modularity	0.388	0.754
Number of Communities	6	62
node	679	315
edge	18618	405
